# Rheumatic Diseases Considered in Regard to Their Nature, Phenomena, Changes, &c., with the Most Rational Treatment of Them, and with Especial Reference to Diet

**Published:** 1842-10

**Authors:** 


### Art. VIII.

*Die rheumatischen Krankheiten, nach ihrem Wesen, &c.* Von Georg Friedr. Greiner, m. d.—*Leipzig*, 1841. 8vo, pp. 215.

*Rheumatic Diseases considered in regard to their Nature, Phenomena, Changes, &c., with the most rational Treatment of them, and with especial reference to Diet.* By Dr. Greiner.—*Leipsic*, 1841.

In a late Number (XXVII.) we gave a pretty full account of an English treatise on rheumatism. We have thought it might be interesting to our readers to know how the same subject is treated by one of our German brethren. With this view we shall principally confine ourselves to an analytic exposition of the contents of Dr. Greiner's volume.

In the preface, Dr. Greiner points out the frequency, Protean varieties, and gravity of rheumatic disease. He reckons that a third part of the race suffers from the malady, which is not, perhaps, to be regarded as an overstatement, if, under rheumatism, are comprehended the various neuralgic affections, which, though not identical with, are nearly allied to, that intractable and formidable disease.

Clear and succinct accounts are given, in succession, of the local affections, as rheumatism of the shoulder, neck, scalp, sacrum, loins; of sciatica, pleurodynia; of rheumatic affections of the muscles of the abdomen and their aponeuroses; of toothach, facial neuralgia, ophthalmodynia: and then of articular rheumatism. So far, we meet with nothing novel. A brief account of rheumatism of the spinal cord follows, which we quote:

"Particular notice is to be taken of rheumatism of the spinal cord, which presents itself with the following characters. The pain is principally in the cervical region. With that is associated internal uneasiness: the look is timid and staring; the eye feverishly bright; the pulse feverish; numbness of the hand, especially of the fingers, with a sense of creeping or stunning. Then follows increased uneasiness, with flushing of the countenance. Symptoms such as those described seize the extremities; in women, the upper, and in men, the lower, are peculiarly affected. The gait becomes unsteady and bending; the feet seem to dangle. If these evils are not speedily arrested, perfect palsy of these parts takes place, which extends elsewhere. The speech becomes stammering, there is insensibility of the skin; the senses decline; the excretions of the bladder and bowels take place with difficulty. The disease may continue some years ere it destroys." (p. 17.)

From page 18 to 23 are some sensible remarks on the nature and character of rheumatic neuralgia, and on the means of distinguishing neuralgia from rheumatism. The presence or absence of intermissions of the pain and of swelling of the affected part, are the conditions on which diagnosis must be grounded. If there are complete intermissions and no swelling, the case may be pronounced one of pure neuralgia. If there are mere remissions, the case partakes more or less of rheumatic complication: and the coexistence of swelling will leave little doubt as to its nature. Neuralgia may be excited by rheumatism extending along either the neurilema or substance of nerves: and this complication is, according to the author, distinguishable from ordinary rheumatism, by the unusual intensity of the pain, by a gnawing sensation, and by a stinging as if of red-hot daggers; these acuter symptoms, however, which mark the neuralgia, remitting occasionally, more especially when the limb or part is allowed repose. Rheumatic neuralgia is rarely accompanied with swelling, and does not, like ordinary rheumatism, change its place, as from the arm to the shoulder, &c., which difference the author explains on the supposition that such metastases or transferences take place more easily between or along the continuous and extended muscles and aponeuroses, than from one set of nerves to another. The nervous system is therefore never so wholly neuralgic as the muscular and fibrous systems are rheumatic.

At page 24, the metastases are treated of, and here we shall quote the author's account of metastases to the head, (nach dem Kopfe.)

"To the internal parts, which are liable to be the seat of rheumatic metastasis, belong all internal muscular structures, in so far as they are provided with aponeurotic and fibrous tissue: further, all parts, which in whole or in part, possess a fibrous, tendinous, or membranous structure. The following are the parts which require particular attention. The dura mater:—To this membrane rheumatic affections transplant themselves more readily and frequently than we should suspect, either from the actual retrocession of the rheumatism from an outward part, as for example, the Galea aponeurotica, the cervical muscles, or other parts of the upper extremities, or from extension of the primary rheumatic affection. In this point of view, the mixed rheumatico-catarrhal affection of the mucous membrane of the throat, tonsils, soft palate, derives importance, inasmuch as from these parts, the rheumatic affection may extend to the *basis cranii,* seize the ligaments of the *atlas* and *epistropheus,* and may thence reach the cavity of the cranium and extend to the *dura mater.* This latter accident is to be suspected when the guttural pain does not reach a true angina, but manifests itself as a mere fleeting, stinging pain of brief duration; while, on the other hand, noises in the ear, remarkable sharpness of hearing, a sort of double hearing, are perceived; and further, when a painful feeling in the nape, especially on motion, arises; also, when, on turning the head from one side to the other, and the neck is bared, and all external noise hushed, a slight crepitation is perceptible in the nape from the motion of the *epistropheus.*" (p. 26.)

He adds that the affection of the dura mater is evidenced, not so much by acute pain, which more marks the affection of the "Galea aponeurotica," as by a slight, dull, uneasiness, not augmented on pressure, or by shaking, or nodding the head, and by a slight trembling of the head in walking, as well as by accesses of giddiness. Thickening of the dura mater, effusion, and, in consequence, increased pain, deafness, double vision, delirium, apoplexy, follow the protraction of such a state of things.

At page 27 the metastatic affections of the viscera of the thorax are referred to, with one unaccountable exception to be noticed presently. At an earlier part of his treatise, (p. 4,) and again here, the author speaks of many obscure and troublesome affections, being really due to rheumatism, either primary or metastatic, though little suspected to be so. There can be no doubt, that, in gouty and rheumatic subjects, a thousand seemingly anomalous symptoms and ailments, are explicable simply from the fact of the rheumatic or arthritic diathesis impressing a peculiar character on every incidental derangement. Rheumatism, for example, and the remark applies to gout, when affecting metastatically a secreting organ, as the kidney in its membranous parts—may betray itself simply or chiefly by a notable derangement of the urinary secretion, and slightly or even not at all by pain. In this way the frequent and often apparently causeless derangements of the urinary secretion of gouty and rheumatic persons (causeless, since they come on notwithstanding the most punctilious attention to diet,) must be explained. In like manner, rheumatic affections of the bronchi and the ramifications of these, may give rise to dyspnœa, and a multitude of pulmonary symptoms of the most perplexing description. The same may be remarked of the metastases to the diaphragm, to the coats of the intestines and to the abdominal muscles and their aponeuroses; all which may lead to the suspicion of visceral disease. In such cases, a knowledge that the patient is rheumatic will solve a thousand difficulties, and facilitate our diagnosis, when otherwise it would be either most unsatisfactory or else impracticable.

The truly unaccountable omission to which we alluded in the beginning of last paragraph, is that of all reference (here at least,) to either metastatic or primary affections of the heart. At a later part, indeed, that important complication is slightly adverted to. It being neither our duty or design to fill up the author's omissions, we content ourselves with simply pointing out this singular oversight.

At p. 40 the question as to the seat of rheumatism is discussed; and the author, while noticing that there is still a diversity of opinion on the subject, adopts the prevailing doctrine, that the fibrous tissue is the one which rheumatism peculiarly affects. As this tissue is the "groundwork" of all animal structures; as it includes muscle, tendon, articular capsule, cellular tissue, (the latter as "the transition-state of the fibrous substance from its fluid into its solid and organized condition,") mucous glandules and follicles, synovial glands, the internal ligaments of joints, the dura-mater as reflected on the medulla oblongata and spinal cord, and accompanying the nerves in their minutest ramifications, the skin—such being the extent of the fibrous tissue, and, by consequence, that of rheumatic susceptibility, it is obvious that the morbid action of rheumatism may pervade the inmost parts and organs. However, it peculiarly affects fibrous tissue, as organized in tendon, ligament, or capsule; and when muscle is seized by it, the lymphatic effusion which characterizes it is, in contra-distinction to the purulent one of common inflammation, never poured out into the substance of the muscle, but always into the cellular tissue. (pp. 40-3.)

The states of the blood and of the nervous system, which are essentially concerned in the production of rheumatism or produced by its development, are next considered. As regards the condition of the former, Dr. Greiner's general conclusion is, (pp. 44-6,) that the fibrous material (faserstoff), "the vital character of which is determined by a magnetic disposition," not only superabounds in the circulation, in rheumatism, but has also, during the continuance of the disease, "a peculiar tendency to quit its soluble state in the blood, and to organize itself." His account of the share which the nerves have in the disease is not very clear, and seems in some measure inconsistent with the views already stated, as to the fibrous tissue being the sole seat of the affection, (with which, indeed the account formerly given of rheumatism of the spinal cord appears also at variance,) since, after noticing that it may be through the medium of the fibrous sheath that the nerves suffer, he remarks, (p. 47,) "that the nerves are also particularly affected, *are often themselves the*

*seat of the disease*, and at all times suffer as organs of sensibility, is beyond all doubt."

At p. 50, the diagnosis between gout and rheumatism is stated to consist in the former being a disease of the periosteum, (?) consisting in an error and excess of the secretive function of the capillary vascular system, by which the bony mass is deposited : rheumatism, on the other hand, is a disease of the aponeurotic system, has its seat in the sheaths and tendons of muscles, and consists in a derangement of the action of the vessels and nerves ministering to the secretion of tendon. The two diseases may run into each other; rheumatism sometimes reaching and affecting the more tendinous kinds of periosteum; and gout, on the other hand, occasionally extending from its original and native seat (the periosteum) to the articular capsules and cartilages. Gouty pain continues irrespective of the limb being at rest, and migrates less readily and to less distances than rheumatic pain does. Gout principally attacks middle and old age, and comes on in spring or in autumn. Rheumatism makes no distinction of ages or seasons.

In the author's etiology of rheumatism we do not find anything new. Cold and moisture and changes of temperature are the chief predisposing causes. Thus it is more frequent in temperate than in either the high or low latitudes. Dr. Greiner is of opinion that certain other diseases, "by producing weakness of the nerves and a change in the quality of the blood," which he elsewhere terms "the rheumatic dyscrasy" of that fluid, predispose to the malady. These diseases are gastric, bilious, mucous, intermitting, and particularly miliary fever. (pp. 54-5.)

We shall not introduce to the reader the abstruse and characteristically German speculation into which the author here departs, (p. 55,) when endeavouring to explain the proximate cause and the essential nature of rheumatism. All of a sudden, Dr. Greiner, the course of whose observations has been hitherto remarkable for its sobriety and rationality, abandons himself to the most fanciful, wild, and, indeed, unintelligible hypotheses. We are told of a "cosmo-telluric life-spirit," of a "nature-life," of a "world-life," "a sun-life." "The earth-life," it is stated, (p. 57,) "strives against the solar-life, in order to gratify its own life-impulse to the development of its inherent creative power: and, in union with the sun-life, to institute a relative independent existence, as child and prototype of the world-life. This upward striving of the telluric to the solar life is realized in the atmosphere!" Our readers will scarcely believe that this unexpected and strange outburst of mysticism and extravagance is from the same pen as that, the calm and judicious remarks of which we have been recording. We do not attempt to convey to the reader any idea of this extraordinary episode in Dr. Greiner's work, for the good reason that it is utterly incomprehensible. Happily Dr. Greiner's return to common-sense is as sudden and complete as his aberration from it; and he proceeds to consider a number of interesting topics, which want of space compels us to pass over. We shall very shortly advert to the therapeutic portion of the volume.

The detail of treatment is sufficiently ample and minute : that relating to acute rheumatism occupying from p. 90 to 113. Our notices must be brief and partial. In the stage of "premonitions," entire rest, avoidance of all violent motion, reclination in bed, or on the sofa, strict diet, mild light nourishment, the eschewing all heating drink and solid food, the use of some gentle salt, as tartrate of borax, in doses of ten or fifteen grains, with the addition of a quarter of a grain of ipecacuan, several times daily, constitute the first parts of the treatment. If the patient be young or middle-aged and plethoric, and the attack be characterized by marked excitement, bloodletting, to the extent of from four to six ounces, may be practised. If an alkaline bath can be taken with convenience, it may be had recourse to. An emetic may be employed, as by its action on the stomachic nerves and through them, on the whole "reproductive nervous system," (by which here and elsewhere the author means the assimilative system,) it may destroy or, at least, modify the rheumatic fever, without either weakening the circulation or augmenting its excitement. The following powder is recommended:

℞. Potassæ Nit. ʒj.

Carb.

Magnes Carb. āā, ʒss.

Sacch. Lactis, ʒvj. M. ft. Pulvis.

A teaspoonful to be taken every two hours; if the patient is of advanced age or debilitated, regulated doses of the spirit of mindererus, as a *scruple*, to which four drops of the tincture of ipecacuan are added, and six or eight grains of Dover's powder at night is the treatment suggested. We have given these details with a view to afford the reader some insight into the author's therapeutic management, in the stage of "premonitions." (pp. 88-9.)

But it is seldom, until the *first* stage of the *formed* disease, that the physician is called in; and, in giving directions as to the management of it, Dr. Greiner quotes a variety of authorities as to the propriety of bloodletting. The general conclusion drawn by him and by them is, that this measure is moderately and cautiously to be resorted to, it being kept in mind that the rheumatic is an inflammation *sui generis*, and differing from ordinary inflammation. A variety of internal treatment, called "cooling" (p. 94), into which nitrate of potass, tartar emetic, carbonate of potass, Glauber salts, tamarinds, enter, is here detailed, but which we have neither space nor leisure to quote. The tincture of aconite, along with that of hyoscyamus, in the almost homœopathic doses, of three or four drops of the former to two of the latter, at night or morning, is also proposed. If narcosis (!) does not follow, the above dose is to be augmented by two drops. If great, but not symptomatic (query, critical?) sweats take place, corrosive sublimate, in the proportion of one grain to ℥j of water, dose fifteen or sixteen drops, three times daily, is alleged to be useful. In the second stage (stadium status), in which there are heavy, but not critical sweats, camphor is recommended in doses of two or three grains four times daily, in an emulsion. Dr. Greiner esteems it an object to arrest the sweats above referred to, and it is partly with this object that camphor is to be employed; but if after a few days those sweats continue, then oleum terebinthinæ or juniper is to be substituted for the camphor in doses of four or five drops.

Here, at last, the author refers to the possibility of metastasis to the heart as well as to the diaphragm. To obviate these mishaps, now apt to take place, he advises, more especially if the pulse of the patient be full and the excitement great, nitrate of potass, along with small doses of tartar emetic; mercury is to be abstained from. When the circulation is quieted, yet some traces of nervous excitement remaining, digitalis, nitre, and chalk are to be given. The digitalis may be united with tartar emetic.

The indication in the third stage (stadium decrementi), is to aid nature in her curative efforts, by promoting secretion and excretion, and by acting on the nervous system, and by regulating the diet. The physician is to make it an object to *produce* sedimentous urine (p. 104), where the excretion does not spontaneously contain deposit, and the diet is to be strictly regulated. For these objects, the following formulæ are recommended :

℞. Sal. ammoniac. depur. ʒij.

Pulv. gum. Arab. ʒiij.

Camphor. grana x. ad ℈j.

Aquæ fontan. ℥iv.

Oxymel. scill.

Syrup. althææ, āā ℥j.

Dose, a table-spoonful every two or three hours. One or two powders of the following combination are to be taken at night: Pulv. aconiti, grana iv.; sacchari albi, ℈iv. Mix most carefully by pounding for many minutes, and divide into four powders. The use of the aconite is to be regulated, as regards quantity and time, by its production or nonproduction of narcosis.

The treatment of the metastasis is chiefly to be managed by anodyne and anti-spasmodic means, as opium, musk, camphor, and in case of the stomach or pericardium being affected, by epigastric frictions of croton oil or tartar emetic.

*Rheumatismus universalis* (p. 112) of the acute kind, is to be treated by lukewarm alkaline baths and assiduous infriction of mercury. Rheumatic tetanus, of which no proper description is given, is usually fatal. According to Neumann's experience, who tried camphor and opium, with cupping-glasses and leeches applied in large quantities along the nape and spine, and the freeest general bloodletting, all remedial measures are utterly futile.

The treatment of chronic rheumatism occupies from page 113 to the end, and we can only very briefly refer to it. The two grand indications are stated to be, 1st, to operate on the nerves, and give them a new tone; and, 2dly, to adopt such measures as shall act beneficially on the blood and free it from the rheumatic dyscracy. The former of these two principal indications is subdivided, 1st, into those means which act directly on the nervous system; and 2dly, into those which act indirectly. The production of narcosis is stated as the first among the direct means, and under this head aconite, stramonium, hemlock, solanum, belladonna, opium, and nux vomica, are enumerated and discussed, in the order here stated. Stoerk's recommendation of aconite is repeated and confirmed in cases of sciatica, rheumatic pains of the limbs, rheumatic headach, cramp of the stomach, megrim, &c. (p. 119.) Stramonium is also recommended, though rather on the experience of others than of the author himself, and great caution is inculcated in its employment. Hemlock is described as acting powerfully on the ganglial system, and to solanum dulcamara the same character is given. Belladonna, opium, and nux vomica, are rather lukewarmly spoken of, and are asserted to have little influence over the peculiar rheumatic derangement of the nerves. Opium is described as not sufficiently "constant and commanding" against the disease.

The metals and earths are the next among the means which act directly on the nerves. Mercury is a powerful agent of this class, operating not only on the nervous, but also on the sanguineous systems, and that in one or all of three ways: 1st, by reducing excitement; 2dly, by determining rapidly to the mucous membrane of the intestines; or, 3dly, by operating on the aponeurotic system, and at the same time improving the quality of the circulating mass. Antimony, iron, copper, arsenic, zinc, are noticed and recommended as occasionally useful. Gold is recommended in the following form and dose:

℞. Auri muriati. gr. vj.
Ext. aconiti
Pulv. herb. aconiti, āā, ℈j.
Pulv. succ. liquir. ℈iv. M.

This mass is to be divided into pills of the weight of one grain each, one pill to be taken morning and evening. The above preparation of gold may also be used in friction, in the proportion of four to five grains to the half ounce of pommade of roses. (p. 136.)

The means by which we act indirectly on the nervous system are as follows: Colchicum, arnica, senega, guaiac, ol. terebinthinæ, camphor, sulphur, sulphuric alcohol, ol. dippelii, cajeput, &c. The *modus operandi* of these is stated to consist in their stimulating the nervous plexuses and functions, producing a salutary influence on the nerves of the muscular and aponeurotic tissues, and an alterative effect on the rheumatic derangement of the nerves, and improving the constitution of the blood. As to colchicum, Dr. Greiner, while admitting its efficacy, is doubtful whether this be due to its veratrine, since this substance acts chiefly on the alvine canal, producing vomiting and purging, and but slightly affecting the urinary secretion. He thinks the oxymel the best preparation of colchicum: arnica, from its property of stimulating the lymphatic system and mucous membrane, and from its "salutary effects on sanguineous stagnation and extravasation," is useful in promoting absorption in rheumatic exudation and swelling. Senega is eligible in similar cases. Guaiac is commended; and of the beneficial operation of the etherial-oleaginous substances, in general, in rheumatism, the following explanation is given. (p. 140.) "Their action is, in some measure, explicable from their constitution; since, consisting in great part of inflammable oxydizable materials, carbon and hydrogen, and containing little oxygen or none at all, they excite in the living frame organo-vital oxydation, and stimulate consequently the nerves of the irritable organ, namely, the nervous plexus of the arterial capillary vascular system, to exalted action." Turpentine is to be employed only after acute symptoms have subsided, and seems to be useful by acting on the skin and on the "nerves of the kidney." Juniper oil operates similarly. Camphor (p. 150) is stated to act as "a moderator, soother, and rectifier of the circulation, particularly of the capillary vascular system." Sulphur is praised, and it is asserted that "it seems, on account of its analogy with hydrogen, and its union with the same, in the organization, to act more on the venous division of the sanguineous system." (p. 153.) Sulphuric alcohol is stated (p. 155) to be a substance worthy of the greatest attention. It is said to be "a powerful means when the extension of the rheumatic affection on internal aponeurotic parts, especially on the serous tissues, as the dura mater, pleura, or peritoneum, is apprehended, since it acts rapidly on the nerves of the reproductive [assimilative] system, and directs a vivifying action to the periphery of the organism;" by which, we presume, morbific action or metastasis to inward parts is prevented by the peripheric stimulation, as just described.

At page 157, the second grand indication is discussed, namely, that which relates to the blood. This, like the preceding, is subdivided into direct and indirect means. The direct are, fat oils, cod-liver oil, sugar, milk-sugar, whey, milk, soda, potass, chalk. The bringing back the blood from its state of rheumatic dyscrasy to its normal condition is affirmed by the author to be as important as restoring the proper function and sensibility of the nerves; and the medicinal employment of the substances first enumerated, is alleged to fulfil the former of these indications, "by being directly taken into the blood, and partly by being there either assimilated as nutriment, or partly by being unassimilated, and thus acting in a therapeutic manner on the sanguineous mass: by these means we aim at directly changing the rheumatic dyscrasy; also at deoxydating, neutralizing the hyper-oxydated, acrid, acid, nerve-irritating, fibrous matter in the blood, at lessening the abnormal coagulability of this fluid, and at diminishing and diluting its too plentiful fibrous material." (p. 158.) As regards fat oil, the author affirms, that while, on being swallowed, it operates mildly and soothingly on the nerves and mucous membrane, and though occasionally apt to disagree with the stomach, affords, when duly digested, strong nourishment, the impregnation of the carbon-hydrate of the fat with oxygen, in the course of its process through the mesenteric glands and the circulation, enriches the blood with coagulable and fibrous material, producing some excitement in irritable organs; which, however, is compensated for by its withdrawing oxygen from the blood, thereby rendering that fluid less acrid and stimulating. It is consequently useful in cases in which "the rheumatic disease is accompanied with poverty of nutrient material in the blood, and at the same time with a peculiar irritability, with emaciation and a slight degree of fever." (p. 160.) Cod-liver oil is moderately praised, and by no means lauded to the extent which Dr. Bennett's late treatise on it would have led us to expect in a German writer.

We are somewhat surprised at the warmth of the eulogium passed on sugar, as one of the direct means of improving the blood. We pass over the chemical and physiological explanation of its modus operandi, and merely notice that the author strongly recommends it in both acute and chronic rheumatism, where sthenic excitement prevails and antiphlogistic means are indicated. To be useful, it must be taken in doses of from one to two ounces, three or four times daily, in syrups, conserves, or plain. Milk may be advantageously combined with it. Whey, milk, soda are noticed and recommended as occasionally and in various degrees and circumstances, useful in the disease.

At p. 90 et seq. the means which operate indirectly on the blood are considered. Rheumatism may attack two classes of persons: either young subjects, plethoric, and with a rich blood, or else those old and debilitated, in whom the circulating fluid is thin and dyscrasic. In the first of these, the salts of mercury, the neutral salts, nitre, sulphuret of potass, common salt, and soda are to be employed to reduce the fibrinous state and coagulable tendency of the blood: in the second, animal food, leguminous vegetables, farinaceous food (mehl speisen), etherial oils, the resins, iron, wine, aromatic spirit, preparations of sulphur are to be had recourse to. (p. 170.)

The *external* means to be employed in rheumatism are divided in the same way as the internal, namely, into those which act directly and indirectly on the nervous and vascular systems respectively. Among those which are alleged to act directly on the nervous system are the following: magnetism mineral and animal, electricity, galvanism, Perkinism, acupuncture, insolation, cold, heat, hot vapour, hot iron. Of both mineral and animal magnetism the author entertains very moderate and therefore, in our opinion, very just expectations. The former sometimes has relieved temporarily, but seldom or never effected radical amendment. A rather more favorable report is given of electricity and galvanism. Perkinism, an American invention or discovery—as the reader may please to consider it—consists in stroking the affected part alternately with an iron and brass needle; a hundred times with each needle separately, and then two hundred times with both together. This operation renders the skin red; but the author does not state whether this effect be not entirely due to the force of the manipulation. It appears to have dropt into disuse. Acupuncture is more confidently spoken of by the author. Both foreign and British (Copland) authorities are adduced in its favour. It is more particularly suited to local rheumatic affections of neuralgic type or complication. Insolation, which is said to act "purely and universally on the nervous system, and to operate as a vital principle in nature, favouring, by constant desoxydation, the plastic of organic life," may be useful with other means. Cold and heat, applied more particularly by the medium of water, are noticed, and their advantages and disadvantages correctly discriminated. Cold applications in rheumatism are to be very cautiously resorted to; since cold and moisture are the principal exciting causes of the disease; and, if unskilfully employed, are exceedingly apt to cause metastases on inward organs. Cold is more particularly indicated in merely local rheumatism of a neuralgic type. As a general prophylactic against that peculiar sensibility of the cutaneous nerves to changes of temperature, which often predisposes to rheumatism, cold is highly useful when employed by sponging with cold water, followed by frictions. Heat is a more safe and more frequently-indicated means. After the first stage of acute rheumatism, it allays pain and soothes both nervous and vascular excitement. In the chronic form of the disease heat acts as a salutary stimulant of the depressed nervous system, and excites the arterial capillaries. The warm water may be impregnated with stimulant or anodyne substances, as may be necessary. Care is to be taken not to persevere in the use of warm applications beyond the proper time, as thereby debility and atendency to lymphatic tumefaction may be caused. Vapour-baths, with camphor, alcoholic liquors, sal-ammoniac, red-hot iron, the moxa, are noticed; but the peculiar cases in which these measures are eligible or justifiable are not at all, or are not clearly pointed out. We merely add, that, as further *external* means of *directly* affecting the nervous system, all the narcotics formerly enumerated may be employed as outward applications.

At p. 188, the indirect external means of operating on the nerves are discussed. These are subdivided into three classes: first, those, such as pitch and mustard-plasters, which immediately act on the cutaneous nerves, producing in the dermoid tissue a peculiar modification of its secernent action, or else some other change which by sympathy reaches the inmost and central parts of the nervous system. Secondly, terebinthinate, balsamic, and alcoholic liniments and plasters, which stimulate both the nervous and sanguineous systems, and influence the processes of secretion and excretion. Thirdly, those means, such as the preparations of mercury and antimony, which correct the derangements, at once, of the sanguineous and nervous systems, and produce, through absorption from the dermoid tissue, specific discharges. (p. 195.)

At from p. 198 to 202, inclusive, sea-bathing, native, mineral, and simple domestic and medicated baths are considered. Great efficacy is justly ascribed to several of the German spas, and the subject is sensibly though briefly discussed. Diet and regimen occupy from 202 to the end. Diet is truly stated to be a matter of great importance in the treatment of rheumatism, since, by means of it, both nervous derangement and sanguineous dyscrasy may be moderated or removed. The author divides diet into the austere, the antiphlogistic, the nourishing, the exciting, and the specific. The circumstances in which the first four descriptions are indicated will easily suggest themselves to the reader. The specific diet consists in the use of saccharo-mucilaginous vegetables, such as strawberries, currants, grapes, carrots, turnips; gruels of barley and groats, cresses, radish, and horse-radish, &c. This section concludes with some remarks on the use of wine, which requires caution, but may be permitted to the old, feeble, and cachectic. Regimen, or the manner of life of the rheumatic subject, should be arranged on the general principle of maintaining the nervous system at the highest tone that can be attained without the use of over-stimulation: this end is to be accomplished by regular exercise, avoidance of fatigue, and the use of all the measures already enumerated.

In treating this volume, our aim throughout has been rather simply to present to the reader the views of one of the most recent German authors on rheumatism, than to make his pages an occasion for critical comment. In concluding, we shall briefly notice the faults of the work. It is deficient in general views; there is no pathological anatomy in it; the metastatic affections of the heart and other internal organs are all but entirely overlooked; there is no reference to the iodide of potass. On the other hand, there are not wanting scientific and acute views and suggestions; the treatment proposed is, on the whole, judicious; and, in so far as the work gives us a notion of the curious and somewhat mystical speculations which prevail at this moment in Germany, in regard to the influence of telluric, solar, atmospheric, and other causes in the production of disease, it will not be uninteresting to the English reader.

